# A Review of Human Coronaviruses’ Receptors: The Host-Cell Targets for the Crown Bearing Viruses

**DOI:** 10.3390/molecules26216455

**Published:** 2021-10-26

**Authors:** Aaya Nassar, Ibrahim M. Ibrahim, Fatma G. Amin, Merna Magdy, Ahmed M. Elgharib, Eman B. Azzam, Filopateer Nasser, Kirllos Yousry, Israa M. Shamkh, Samah M. Mahdy, Abdo A. Elfiky

**Affiliations:** 1 Biophysics Department, Faculty of Science, Cairo University, Giza 12511, Egypt; ibrahimmohamed@gstd.sci.cu.edu.eg (I.M.I.); FatmaGalal_PG@alexu.edu.eg (F.G.A.); Merna.hakiem@gmail.com (M.M.); Ahmed.bph32@gmail.com (A.M.E.); 2 Physics Department, Faculty of Science, Alexandria University, Alexandria 21519, Egypt; 3 Physics Department, Medical Biophysics Division, Faculty of Science, Helwan University, Cairo 11511, Egypt; emo.w161@gmail.com; 4 Biochemistry Department, Faculty of Science, Cairo University, Giza 12511, Egypt; filopateernasser44@gmail.com; 5 Faculty of Medicine, Cairo University, Cairo 11511, Egypt; kirllosyousry536@gmail.com; 6 Independent Researcher, Giza 12511, Egypt; esraa.m.ahmed@std.agr.cu.edu.eg; 7 National Museum of Egyptian Civilization, Ain Elsira-Elfustat, Cairo 11511, Egypt; samah_elmahdi@yahoo.com

**Keywords:** human coronavirus, cell receptor, viral entry, spike, SARS-CoV-2, COVID-19

## Abstract

A novel human coronavirus prompted considerable worry at the end of the year 2019. Now, it represents a significant global health and economic burden. The newly emerged coronavirus disease caused by the severe acute respiratory syndrome coronavirus-2 (SARS-CoV-2) is the primary reason for the COVID-19 global pandemic. According to recent global figures, COVID-19 has caused approximately 243.3 million illnesses and 4.9 million deaths. Several human cell receptors are involved in the virus identification of the host cells and entering them. Hence, understanding how the virus binds to host-cell receptors is crucial for developing antiviral treatments and vaccines. The current work aimed to determine the multiple host-cell receptors that bind with SARS-CoV-2 and other human coronaviruses for the purpose of cell entry. Extensive research is needed using neutralizing antibodies, natural chemicals, and therapeutic peptides to target those host-cell receptors in extremely susceptible individuals. More research is needed to map SARS-CoV-2 cell entry pathways in order to identify potential viral inhibitors.

## 1. Introduction 

Human coronaviruses (CoVs) are a new type of virus (order *Nidovirales*) identified in the mid-1960s and classified taxonomically under *Coronaviridae* family and *Coronavirinae* subfamily [1,2]. Coronaviruses are given this name for the crown-like spikes on their surface and are classified, based on their genetics, into four main groups known as alpha, beta, gamma, and delta coronaviruses. The majority of gamma coronaviruses and delta coronaviruses affect birds, whereas alpha coronaviruses and beta coronaviruses infect rodents and bats [3]. There are seven known coronavirus strains that can infect humans: 229E and NL63—alpha coronaviruses; OC43, HKU1, MERS-CoV, SARS-CoV, and the newly identified SARS-CoV-2—beta coronaviruses [4]. Sometimes coronaviruses that infect animals can also make people sick and turn into human coronaviruses, as in cases with SARS-CoV, MERS-CoV, and the new SARS-CoV-2 [5,6]. The infectious bronchitis virus (IBV) was the first CoV discovered, and it primarily infected the respiratory systems of chickens. On the other hand, the first two human coronaviruses identified were HCoV-229E and HCoV-OC43, which cause common cold symptoms in people [7,8].

Severe acute respiratory syndrome (SARS), which is a viral respiratory disease caused by a SARS-associated coronavirus (SARS-CoV), was first identified in 2003 during an outbreak that emerged in China and then spread to more than 30 countries, resulting in a fatality rate of nearly 10% (774 deaths out of 9098 cases), turning the world’s attention to human coronaviruses [9,10,11]. Since then, several other HCoVs were identified; nearly 30 strains were found. The first HCoV strain identified was B814 that was isolated in 1965 [12]. In the post-SARS era, several other HCoVs strains appeared, including HCoV-NL63 in 2004, HCoV-HKU1 in 2005, and 229E and OC43 between 2003 and 2005 [13], which caused mild to moderate upper-respiratory tract illness in humans, resulting in approximately 15–30% of common cold cases [8]. Later in 2012, another human coronavirus with a higher fatality rate (35%) invaded the Middle East and spread to other countries, which was then named the Middle East Respiratory Syndrome coronavirus (MERS-CoV) [14,15,16,17]. Recently, at the end of December 2019, specifically in Wuhan, China, a new coronavirus was discovered in a number of patients suffering from severe pneumonia, resulting in a new disease called coronavirus disease of 2019 (COVID-19), which turned out to be a new type of human coronaviruses and was given the name SARS-CoV-2: severe acute respiratory syndrome coronavirus 2 [18,19,20,21].

Different host-cell receptors are utilized by viral proteins to recognize host cells, such as integrins, angiotensin-converting enzyme 2 (ACE2), sialic acid receptors, dipeptidyl peptidase 4 (DPP4), and glucose regulated protein 78 (GRP78). The purpose of this review is to provide a comprehensive overview of the various human coronaviruses strains that have been identified, and to highlight the multiple human host-cell receptors used by viruses to enter cells. It is important to understand how this group of viruses can recognize and enter human cells. This may help to prevent future epidemics and pandemics caused by novel human coronaviruses.

## 2. Coronavirus Structure

Coronaviruses have a single-strand of positive-sense RNA of up to 31.7 kb and a capped 5′-end [22]. They are enveloped. Their sizes range from 80 to 120 nm. They can be spherical or pleiomorphic in shape. CoVs have the largest viral RNA genomes, ranging from 26 to about 32 kb [23]. They have six to ten open reading frames (ORFs). The first ORF encodes the replicase proteins and takes up nearly two-thirds of the genome’s length. The last third is responsible for encoding the structural proteins in a specific order: hemagglutinin esterase (HE) (in some strains), envelope (E), spike (S), nucleocapsid (N), and membrane (M). The genome can be found inside a lipid bilayer and packaged in a helical nucleocapsid [22]. The rest of the structural proteins (S, E, and M) can be found on the virion’s envelope, include some coronaviruses’ HE protein. The S protein is a glycosylated protein forming homotrimer spikes, and these viruses are known as coronaviruses because they resemble royal crowns when viewed under an electron microscope. [24,25]. The spike protein is responsible for the attachment and entry of the virus into the host-cell. M and E proteins take part in the viral assembly [26,27].

## 3. Coronaviruses’ Spike Proteins

The key element which is crucial in HCoV infection is the spike (S) protein surrounded by a lipid bilayer on the surface of the virus, from which trimer class I transmembrane glycoproteins protrude [28,29]. The spike (S) protein of SARS-CoV-2 mediates the recognition of the host-cell receptors and facilitates the cell attachment and the cell membrane fusion during the viral infection [30,31,32]. The trimeric S protein is located on the virion’s surface and acts as the basic unit for the host-cell receptor recognition, and it is composed of two components, S1 and S2 subunits [33,34].

Subunit S1 of the spike protein contains the receptor-binding domain (RBD), which is mainly responsible for the cell recognition and the binding of the virus to the host-cell receptors, such as angiotensin-converting enzyme 2 (ACE2) in both SARS-CoV and SARS-CoV-2. The subunit S2 domain contains hydrophobic heptad repeats (HRs) domains HR1 and HR2 that mediate the blending of the viral cell membrane and produce a six-helical bundle [35,36,37]. The spike protein possesses an extracellular N-terminus (180–200 kDa) transmembrane (TM) domain that is attached to the membrane of the virus and a short intracellular C-terminus segment [35,37].

With a few exceptions, in most alphacoronaviruses and betacoronaviruses, the virions hold a spike protein that is uncleaved, whereas in some beta- and all gamma- coronaviruses, the spike is cleaved between the S1 and S2 domains by the Golgi-resident host protease, furin [11].

**Figure 1:**
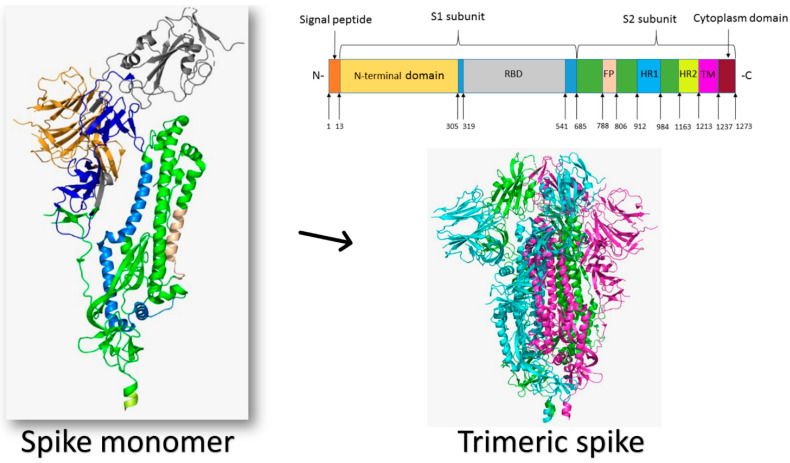
The structure of the SARS-CoV-2 spike protein (PDB ID: 6VXX). The monomeric spike is shown as a cartoon colored by its different domains from its N-terminus to the C-terminus. The spike protein is mono-trimeric, forming a crown-like structure over the virion.

Structurally, the viral spike protein has a total length of 1273 amino acids. It has a signal peptide (1–13) at the N-terminus. The S1 subunit (14–685) and the S2 subunit (686–1273) are in charge of receptor binding and cell membrane fusion, respectively. The N-terminal domain (14–305) and the receptor-binding domain (RBD, 319–541) are found in the S1 subunit; the S2 subunit contains the fusion peptide (FP) (788–806), heptapeptide repeat sequence 1 (HR1) (912–984), HR2 (1163–1213), the TM domain (1213–1237), and the cytoplasm domain (1237–1273) [38]. Figure 1 illustrates the structure of the SARS-CoV-2 spike protein.

The virus uses this homotrimeric class I glycosylated fusion spike protein to enter the host cell. The homotrimer spike protein is found on the surface of the virion, mainly (97%) in the prefusion isoform. Binding to the host cell destabilizes the prefusion homotrimer by shedding off the S1 subunit, and allows for the transition of the S2 to the highly stable postfusion conformation [28,39,40]. This state is grouped into open and closed substates depending on the orientation of RBD relative to the trimeric S protein. One RBD (55%) or two RBDs (14%) may be in the open conformation, which enables the virus to recognize host-cell receptors, facilitating its attachment and hence entrance into the host cell. On the other hand, approximately 31% of the prefusion isoform is found in the closed configuration when the N-terminal domain (NTD) of the S protein covers the RBD [41]. Once the virus recognizes the host-cell receptor, extensive structural rearrangement of the trimeric spike protein occurs, allowing the virus to integrate into the host cell’s membrane, a postfusion isoform process. The virus’s spikes are covered with polysaccharide molecules to hide them from the immune system detection during their entrance [35,42]. The glycosylation of the spike is critical for designing antibodies against the human coronaviruses, as sugars cover most of the exposed spike protein. On the other hand, the carbohydrate moieties over the spikes may be targeted by carbohydrate inhibitors such as chitosan derivatives [43].

Coronaviruses get their name from the bulbous, crown-like halo formed by the S protein trimers around each viral particle. The subunits S1 and S2 create the bulbous head and stalk, respectively, according to structural modeling of coronavirus S protein monomers [44]. At the atomic level, cryo-electron microscopy images determined the SARS-CoV-2 trimeric S protein structure, and revealed various conformations of the S protein RBD domain in open and closed states and their corresponding functions [30,39]. For a coronavirus to enter a host cell, an interaction between specific host-cell receptors and the viral S protein is required. The viral envelope is then bonded to the host cell, allowing the nucleocapsid to enter the host cell. The viral S protein is a type I transmembrane protein ranging from 1160 to 1400 amino acids in length, and it can have 21 to 35 N-glycosylation sites [11].

The viral S protein’s two terminal regions have different roles in the virus’s entrance process. The N-terminal, known as the S1 domain, is responsible for the virus binding with the host cell. On the other hand, the C-terminal, known as the S2 domain, is responsible for the virus’s fusion with the host cell’s membrane. The S1 domain can further be subdivided into N and C-termini, which in turn bind to different host-cell receptors [11]. The viral S protein is synthesized as a single large polypeptide; however, to facilitate its entrance into the host cell, a two-step process is needed. The first step involves the proteolysis of the S1–S2 domain junction, which removes some of the structural constraints from the S2 domain [45]. Next, a second cleavage occurs at the S2’ site that exposes the FP—that is, the functional fusogenic element of the spike, enabling its insertion into the host cell’s membrane [44,46].

### 3.1. The Human Coronaviruses 

Betacoronavirus is one of four genera of coronaviruses that require humans and other mammals as hosts to replicate. Coronaviruses are of clinical importance, as they infect people, resulting in common colds and severe acute respiratory syndrome. The following sections provide a summary of the four most common types of HCoV identified. The history and progression of human coronaviruses are discussed, along with the various host-cell receptors that can be involved in the viral entry mechanism.

### 3.2. HCoV-229E Strain

**Figure 2:**
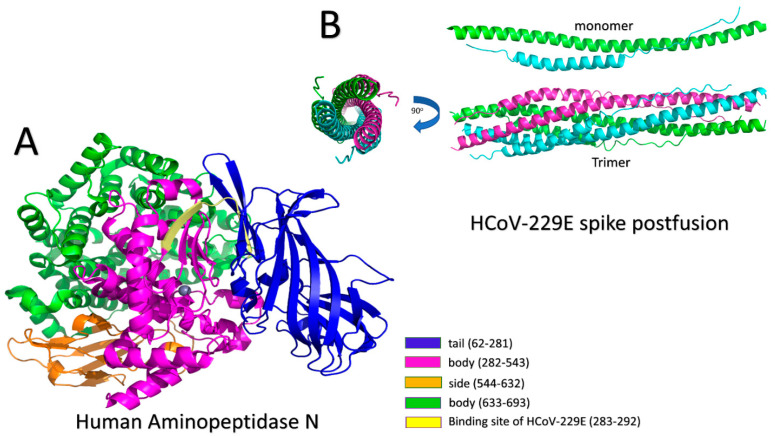
(**A**) Human aminopeptidase N (APN): different parts and the binding site with HCoV-229E (PDB ID: 5LHD). Blue: tail (62–281), pink: body (282–543), orange: side (544–632), green: body (633–693), and yellow: binding site of HCoV-229E (283–292). A Zn^+2^ ion is in the middle. (**B**) A monomer of the postfusion core structure of HCoV-229E (top). HR1 (green cartoon) consists of 24 alpha helices. HR2 (cyan cartoon) contains two conformations, including 9 alpha helices (from amino acid 1067 to amino acid 1098) and extended conformation in both cases. The postfusion core (trimer) structure of HCoV-229E (bottom) is shown from side and top views with 90° rotation angle.

The first human coronavirus identified was the 229E strain, which was isolated and sequenced in the mid-1960s [47]. The 229E strain usually causes mild to moderate upper-respiratory tract illnesses, including common cold symptoms such as cough, nasal congestion, and headache [48]. However, children, the elderly, and immunosuppressed patients are at risk of severe infections [49]. HCoV-229E utilizes mammalian aminopeptidase N (APN) as its entry receptor to invade human cells [11,50]. APN, as shown in Figure 2, belongs to the M1 family, a zinc-dependent ectoenzyme that can be found in the nervous system and on the surfaces of renal and intestinal epithelial cells [51,52]. APN has been verified to play a key role in tumorigenesis, angiogenesis, cell migration, and metastasis; and has been documented as a major target for drug development [53,54]. The APN’s main function is to control the cleavage of neutral peptides from the N-terminus of various oligopeptides. Moreover, it is used as a receptor for some alpha coronaviruses, such as feline coronavirus (FCoV) serotype 2, HCoV-229E, transmissible gastroenteritis virus (TGEV), and canine coronavirus (CCoV) serotype 2 [11].

HCoV-229E binds to mammalian APN amino acids at positions ranging from 283 to 292, as illustrated in Figure 2A [55]. For the binding to occur, the receptor-binding domain (RBD) should be in the active (up) state, which helps with reaching the postfusion form [56,57]. In the prefusion stage, the S1 domain can be seen above the S2 domain, exerting some constraints [45]. The next step for CoV-229E entrance is membrane fusion, which is performed with the help of the S2 domain of the spike protein. CoV-229E S protein is a class I fusion protein, which means that the C-terminus is characterized by the formation of an α-helical coiled-coil structure [37]. The S2 domain of the spike protein has two heptad repeats (HR1 and HR2), a fusion peptide (FP) and transmembrane helix (TM), arranged in the following order: FP–HR1–HR2–TM [45]. The fusion core in CoV-229E is similar to that in other CoVs, such as MERS-CoV, SARS-CoV, and NL63; however, CoV-229E has longer heptad repeats (HR1 and HR2). Therefore, the hydrophobic core packing and the interfaces between the two HRs are different from those of MERS-CoV and SARS-CoV [45]. The postfusion core structure of HCoV-229E was solved at a resolution of 1.86 Å (PDB ID: 5YL9) [45]. A fusion protein, including HR1 (910–988) and HR2 (1162–1206) regions, with a flexible 6-residue linker (L6, SGGRGG) in between, was developed for the analysis of crystallographic research trying to understand the structural core of the interactions between HR1 and HR2 regions of SARS-CoV-2 [45,58]. Similar linkers were used with other fusion core structures of SARS-CoV-2 and were found not to affect the interaction between HR1 and HR2 [59,60]. According to their structures, HR1 was found to form an alpha-helix of 24 turns, and HR2 had two conformations. They were each folded into nine-turn alpha-helices from residues 1067 to 1098, with residues on both sides forming an extended conformation as illustrated in Figure 2B [45]. The fusion core is composed of a trimer of HR1 and HR2, which are organized so that the three HR1 regions are inside the hydrophobic core. At the same time, each HR2 region is placed between two adjacent HR1 regions [45]. This formation resulted in six helices of 128 Å length and 32 Å diameter. The interactions between HR1 and HR2 are mainly hydrophobic. The side chains of residues P1053, L1055, V1057, Y1060, L1065, L1067, L1074, L1081, L1088, I1095, V1100, L1102, and W1104, which reside in HR2, are buried in the pockets formed from the 3HR1 hydrophobic core [45]. On the other hand, another packing type was found in the areas where the surface of the hydrophobic core is flat. In this type, residues E1070, I1071, K1077, S1078, T1084, V1085, L1091, I1092, T1098, and L1099 pack nearly half of their side-chains’ solvent-accessible surface area (SASA) against HR1 flat regions [45].

### 3.3. Severe Acute Respiratory Syndrome Coronavirus (SARS-CoV)

Severe acute respiratory syndrome coronavirus (SARS-CoV) caused an infectious disease that originated from Southern China at the end of 2002, resulting in high mortality and morbidity rates; and within six months after its discovery, the disease affected 8000 individuals and killed 800 [61]. The infection was caused by the SARS-associated coronavirus (SARS-CoV), which caused respiratory and gastrointestinal problems in both humans and pets [62]. SARS-CoV has been reported to infect humans and several other animals [63]. Efforts to clarify the origin of SARS-CoV, hypothesizing a wild animal reservoir, have not been confirmed, and the absolute origin of the virus is still unknown. However, extensive investigations led to the identification of the presence of a coronavirus in civet cats which has shown more than 99% sequence identity to the human SARS-CoV sequence [63,64].

Coronavirus research analysis suggests cytocidal and immune-mediated impacts on cells which can cause cell lysis or apoptosis and can also cause cellular fusion, leading to syncytia. The formation of syncytia in the lung tissue has been identified in SARS-CoV research studies, which has been linked to tissue fibrosis [65].

SARS-CoV attacks alveolar epithelial cells of type 1 and type 2, and the differentiated bronchial epithelial cells of the lung, resulting in an alveolar epithelial cell desquamation. SARS-CoV can also attack the intestinal epithelium of the gastrointestinal tract, causing diarrhea (the virus was observed in a patient’s stool). Additionally, patients infected with SARS-CoV have shown elevated pro-inflammatory cytokines and chemokines in their blood [66].

The coronavirus family members are positive-sense enveloped viruses with single-stranded RNA genomes 30–32 kb in length, and consist of five open reading frames (ORFs) [67], with a 5′ frameshifted polyprotein (ORF1a/ORF1ab), which represents about two-thirds of the genome. Four 3′ structural proteins—namely, spike (S), membrane (M), envelope (E), and nucleocapsid (N) proteins, are shared in coronaviruses, but not the hemagglutinin-esterase (HE) protein. It is found in some coronaviruses [63,68].

For CoVs to deliver their nucleocapsids into host cells, CoVs rely on envelope fusion with the host cell’s membrane. The spike glycoprotein (S) facilitates the virus’s entrance. Therefore, the S proteins mediate both the binding and the fusion of the virus with the host-cell receptors, where the S protein is cleaved by furin or other proteases into S1 and S2 domains, containing 666 and 583 amino acid residues, respectively [11,69]. The S proteins bind to the targeted host-cell receptors, commonly located in the N-terminal region of S1. The fusion process is mediated by the S2 domain of the spike protein [63,69].

SARS-CoV has shown an affinity to bind to angiotensin-converting enzyme 2 (ACE2) through the S1 domain of its S protein [69]. The ACE2 structure shows a hook-like N-terminal peptidase domain containing an active site at the base of the groove. The ACE2/SARS-CoV S complex is composed of 19 to 615 residues of the N-terminal peptidase of ACE2 and 323 to 502 residues of the receptor-binding domain (RBD) [69].

The receptor-binding domain of ACE2 contains a core and an extended loop. The former is a five-stranded anti-parallel β-sheet connecting three short α-helices. In addition, there are nine cysteines in the chymotryptic fragment. Only six of them are involved in disulfide bonds, and the remaining cysteines are disordered [69,70]. The extended loop subdomain is located at the edge of the core and is formed by two-stranded β-sheets. In contrast, the crystal structure of 306–527 residues of the receptor binding S1/ACE2 complex has revealed that a loop within 424–494 residues of RBD is responsible for the interactions of ACE2, and it is called the receptor-binding motif (RBM) [71]. The interaction between the SARS-CoV-2 RBD and ACE2 has been identified using molecular modeling analysis, and the results found some potential residues involved in the interaction. The SARS-CoV RBD and ACE2 binding analysis showed a total of SARS-CoV RBD 16 residues in contact with 20 ACE2 residues that interact with two different RBDs, revealing 17 residues that have been shared between both interactions, mostly located at the N-terminal helix [72]. Six tyrosine-rich (6 Tyr) residues are involved in direct binding to the ACE2 receptor [69].

Jeffers and colleagues showed that the CD209L (L-SIGN) receptor, present on the surfaces of macrophages and dendritic cells, can serve as an alternative gate for infectious SARS-CoV instead of ACE2 [73]. CD209L is a type II transmembrane glycoprotein that contains 376 amino acids and belongs to the C-type lectin family. It comprises a transmembrane domain, a short cytoplasmic tail, an extracellular stalk, and a sizeable C-terminal carbohydrate-recognition domain (CRD). The CRD domain of the CD209L is the binding site of the SARS-CoV spike protein [73].

The CoV entry process is achieved by either the binding of the viral envelope’s contents to the membrane of the host cell or by receptor-mediated endocytosis. The fusion of the two is mediated by the S2 region of the viral S protein [66]. Once the viral RNA enters the cytoplasm, an RNA-dependent RNA polymerase translated from the plus-stranded viral genomic RNA makes a negative strand template from which it synthesizes a series of 3′ co-terminal nested genomic mRNAs. The virus growth cycle is about 10–12 hours in the cytoplasm. Newly formed virions bud into the rough endoplasmic reticulum and accumulate into intracytoplasmic vesicles. The formed virions are carried by the Golgi apparatus to the plasma membrane, where they are released by exocytosis. Viral infection may result in cell lysis or fusion, leading to the formation of syncytia [66]. Extensive research is needed to investigate the complex mechanism of the viral binding and the entry method. A focus on genomic analysis of the virus transcription and translation is also needed. Such research will shed some light on potential targets for developing anti-SARS-CoV treatments and new therapeutic strategies.

### 3.4. Middle East Respiratory Syndrome Coronavirus (MERS-CoV)

The Middle East Respiratory Syndrome Coronavirus (MERS-CoV) is a beta-coronavirus causing severe lower respiratory tract infections. Its mortality rate may be as high as 35% [16]. A MERS infection was first announced in Saudi Arabia in September 2012. However, health officials reported that the first reported cases of MERS-CoV were in Jordan in April 2012, where the virus spread between people through close contacts, and 80% of the cases were reported in Saudi Arabia [74]. The pandemic of MERS infection reached neighboring countries in 2012, including Bahrain, Qatar, Kuwait, Jordan, and Tunisia [75], through infected travelers visiting Saudi Arabia. Later, the outbreak further spread to 27 countries across the globe, reaching North Africa, Southeast Asia, Europe, and the United States of America [76]. During the pandemic, healthcare facilities were required to implement strict contact restrictions, including patient isolation and the use of personal protective equipment (PPE) such as gloves, N-95 respirator masks, and gowns [17,77]. By January 2020, there had been 2519 confirmed cases and 866 deaths as a result of the virus and its consequences [78].

Symptoms observed in the reported cases of MERS-CoV infection included cough, headache, fever, rhinorrhea, shortness of breath, gastrointestinal problems, vomiting, nausea, myalgia, and weakness [79]. However, some confirmed cases were asymptomatic [80]. Patients with serious infections suffered from respiratory failure. Those between the ages of 50 and 59 had the highest risk of infection and mortality, and adults between the ages of 30 and 39 had a moderate risk of infection (the highest of any age group). As immunocompromised patients with diabetes, kidney failure, and lung disease are more susceptible to such infections, MERS-CoV caused sever outcomes among them [81].

Recent research findings indicate that dromedary camels and bats operate as reservoirs for MERS-CoV and can transmit the virus to humans [76,79,81]. MERS-CoV can spread from person to person via airborne particles or infectious respiratory droplets, which are spread by direct contact with infected patients. Transmission of MERS-CoV has been reported in hemodialysis systems, intensive care units (ICUs), and other inpatient facilities in a variety of healthcare settings [82]. The virus is transmitted through humans by inhaling aerosol droplets transferred by coughing or sneezing. Contact with infected surfaces, equipment, and gadgets causes indirect transfer. MERS-CoV has an incubation period ranging from 5.2 to 12 days [76].

MERS-CoV has four structural proteins, S, E, M, and N, but it lacks the HE protein. The spike (S) protein is important for host-cell receptor binding, cell fusion, and infection. Coronaviruses enter host cells using an envelope-anchored trimeric spike protein which binds to a host receptor before the viral and host membranes fuse. The spike protein is made up of receptor-binding (S1) and membrane-fusion (S2) subunits. The S1 subunit contains an N-terminal domain (NTD) and a C-terminal domain that serve as receptor-binding sites (RBD) [83]. The envelope (E) protein is found mostly in the virus’s intracellular membranes and is essential for viral assembly, budding, and intracellular trafficking. The membrane (M) protein is a component of the viral envelope that interacts with other viral proteins to aid in virus morphogenesis and assembly [84]. The nucleocapsid (N) protein protects viral RNA by coating it and participating in the transcription process [85].

As a functioning cellular receptor, MERS-CoV can use dipeptidyl peptidase 4 (DPP4), also termed cluster of differentiation 26 (CD26) [86,87]. MERS-CoV S RBD has a core subdomain that is comparable to SARS-CoV S RBD, and a unique receptor-binding motif (RBM) that interacts with the DPP4 receptor [88,89]. MERS-CoV can also recognize other molecules, making it easier for them to attach to cells and enter cells. Carcinoembryonic antigen-related cell adhesion molecule-5 (CEACAM5) and tetraspanin CD9 (tetraspanin CD9), for example, have been found to be components that promote viral entrance into susceptible cells [90,91]. Furthermore, glucose regulated protein 78 (GRP78) has been identified as a MERS-CoV spike protein attachment factor that promotes viral entry in the presence of DPP4. In addition to its role in viral entry, GRP78 also plays a function in MERS-CoV replication. Although knocking down GRP78 resulted in a reduction in virus replication, it was less than the drop in virus replication that occurred when DPP4 was downregulated. After MERS-CoV enters the cell, cell-surface GRP78 (CS-GRP78) is upregulated, facilitating the virus’s binding [92].

Spike requires proteolytic cleavage to activate membrane fusion. This occurs once the MERS-CoV S protein engages the host-cell receptor DPP4 via its receptor-binding site [93,94]. Depending on the presence of host-cell proteases, MERS-CoV can enter the cell via two different pathways. Serine proteases such as transmembrane protease serine subtype 2 (TMPRSS2) activate the plasma membrane route, whereas cysteine proteases such as cathepsin L activate the endosomal pathway in the absence of cell-surface proteases to complete viral entry [93,95]. In response to the varied virus trafficking timings prior to membrane fusion, the MERS-CoV point of entry is connected with entry kinetics. Viral entry at the plasma membrane is termed “early,” and entry through endosomes is called “late” [91,94,95].

The cell membrane fusion is directed by a sequence of conformational changes in the S protein. S2 separates from S1 during cell fusion. S2’s two heptad repeat regions, HR1 and HR2, interact to produce a 6-helix bundle (6-HB) fusion core. The virus–host-cell membrane fusion is then enabled by exposing a hydrophobic fusion peptide introduced into the host membrane [96,97].

### 3.5. Severe Acute Respiratory Syndrome Coronavirus 2 (SARS-CoV-2)

The World Health Organization (WHO) named the coronavirus disease of 2019 (COVID-19) after new incidents of pneumonia were discovered in Wuhan City in December 2019 [29]. COVID-19 disease statistics as of 4 June 2021, showed about 173,155,362 confirmed cases and 3,723,403 announced deaths in 220 countries and territories around the world [98,99]. SARS-CoV-2 is a betacoronavirus with an enclosed, single-stranded, positive-sense RNA genome [100]. SARS-CoV-2 is 98% genetically similar to bat coronavirus RaTG13, and its closest human coronavirus relative is SARS-CoV, which has 79% genetic similarity [101]. Fever and a dry cough are common symptoms of a COVID-19 infection. Shortness of breath, myalgia, joint discomfort, loss of taste and smell, diarrhea, nausea, and progressive cough symptoms are also common [102]. SARS-CoV-2 causes modest symptoms in the general population when compared to other infections [103]. However, patients suffering from other comorbidities such as asthma, diabetes, cardiovascular disease (CVD), and other chronic diseases are more susceptible to developing severe COVID-19 symptoms or dying [104,105].

The S protein of many HCoVs is cleaved at the interface between the S1 and S2 subunits, which are non-covalently bonded in the pre-fusion conformation. Multiple conformational states of the SARS-CoV-2 S protein were identified using cryo-EM 3D structure data, each corresponding to a different organizational pattern of the receptor-binding domains within the S1 unit. According to recent findings, the SARS-CoV-2 S protein has been identified as a trimer. One of the three RBDs rotated up in a receptor-accessible conformation is the predominant state of the trimer [106]. It has a 160 Å ectodomain with a triangular cross-section, similar to SARS-CoV S (Figure 1).

The SARS-CoV-2 S1 subunit, like other betacoronavirus S glycoproteins, has a V-shaped structure. The three human angiotensin-converting enzyme 2 (ACE2) recognition motifs are buried at the interface between the S1 and S2 subunits in the closed state [107]. Heterogeneous N-linked glycans emerging from the trimer surface are densely coated with the spike glycoproteins. These oligosaccharides are involved in S folding, host protease priming, and antibody recognition modulation [30]. The crystal structure of the SARS-CoV-2 S protein’s C-terminal domain (CTD) in combination with human ACE2 suggests a binding mode similar to that of SARS-CoV. The major residue substitutions in SARS-CoV-2 S CTD show a slight increase in the affinity for receptor binding when compared to the SARS-CoV binding interface. Despite the fact that SARS-CoV and the new SARS-CoV-2 have more than 70% sequence identity in the S protein and engage ACE2 via CTD, the two viruses’ CTDs have been determined to be antigenically distinct. None of the monoclonal or polyclonal antibodies that target the SARS-CoV S1 domain recognize the SARS-CoV-2 S protein [29].

## 4. Coronavirus Cellular Receptors

### 4.1. ACE2 Receptor

Coronaviruses enter host cells by receptor-mediated endocytosis, which is the most common mechanism of entrance [108]. SARS-CoV-2, like SARS-CoV, has been demonstrated to enter the host cell via the ACE2 receptor, whereas its entry mechanism relies on the cellular transmembrane protease serine 2 (TMPRSS2). TMPRSS2 is required for SARS-CoV-2 infection of lung cells [101,109]. The ACE2 receptor is a membrane protein that functions as an inhibitor of angiotensin II (Ang II). It also functions as a receptor for SARS-CoV, and it is internalized with the virus, resulting in a decrease in cell-surface ACE2 and an increase in serum Ang II. As a result, the downregulation of ACE2 protects against the development of acute respiratory distress syndrome (ARDS) and lung injury [101]. 

The renin–angiotensin pathway is involved in the disease pathophysiology of hypertension, diabetes, and cardiovascular disease [110,111]. As a common byproduct of the renin–angiotensin mechanism, Ang II is a powerful vasoconstrictor that induces hypertension and is linked to complications in cardiovascular diseases and diabetic patients [112,113]. Ang II production is counterbalanced through ACE2 that SARS-CoV-2 uses to enter and infect alveolar epithelial cells [110]. Activation of the renin–angiotensin pathway has also been suggested to predispose patients’ comorbidity to severe COVID-19 symptoms [111,114]. The indirect effects of Ang II receptor blockers (ARB) and ACE inhibitors in patients with CVD, and associated comorbidities could theoretically enhance the docking site for SARS-CoV-2, resulting in severe COVID-19 symptoms. [115,116].

Clinical research assessed the association of ARBs and ACE with severe COVID-19 infection in CVD patients. The results showed that suspending treatment (*n* = 334) or continuing treatment (*n* = 325) had no effect on the COVID-19 mortality rate, and the authors suggested that the indirect increase in ACE2 does not elevate the intracellular viral load and is not subject to severe COVID-19 infection [114]. When compared to other COVID-19 groups, patients with pre-existing CVD showed an increased death rate and were more likely to develop severe COVID-19 symptoms [117]. Table 1 illustrates the high mortality rate with pre-existing CVD in COVID-19 patients. As a result, regardless of ACE2 expression, SARS-CoV-2 can use additional receptors to enter the cell and infect it.

SARS-CoV-2 cell entry is dependent on the S protein binding to cellular receptors via the S1 subunit; following that, it is primed by host-cell proteases, resulting in S protein cleavage at the S1/S2 and S2-sites, permitting viral and cellular membrane fusion [101]. The SARS-CoV-2 S/ACE2 interface has been explained at the atomic level, and ACE2 utilization efficiency is a critical determinant of SARS-CoV-2 transmissibility [128,129]. SARS-CoV-2 S is primed for cell entry by the cellular TMPRSS2, and serine protease inhibitors prevent lung cell infection [101]. For S protein priming in cell lines, SARS-CoV-2 can use the endosomal cysteine proteases cathepsin B and L (CatB/L) and the serine protease TMPRSS2. Both proteases must be inhibited for the viral entrance to be completely blocked. However, only TMPRSS2 activity is required for viral spread and pathogenicity [101,128].

The expression levels of ACE2 can differ between children and adults. Previous studies have shown that ACE2 is more prevalent in well-defined ciliated cells and that gender influences expression levels, with men having higher levels than women [130,131]. Type I cell-surface glycoprotein is produced by the ACE2 gene, which is about 100 kDa with 805 residues. A 17-residue N-terminal signal peptide precedes the peptidase domain (PD) (residues 19–615), which contains the HEXXH zinc-binding metalloprotease motif. [128]. The C-terminal collectrin domain (residues 616–768) features a ferredoxin-like fold neck (residues 615–726), followed by a 22-residue hydrophobic transmembrane region, and a 43-residue intracellular section. The HEXXH histidine motif is found in a wide range of zinc-dependent metalloproteases. [132]. The S1 region of the S protein in SARS-CoV-2 binds to the peptidase domain (PD) of ACE2 via TMPRSS2 facilitating viral entry.

### 4.2. Integrin Inhibitory Role in SARS-CoV-2 Receptor Targeting

The spike protein’s structure is critical for recognizing the ACE2 receptor on the host cell [133]. ACE2 binds with its receptor-binding motif (RBM) to the receptor-binding domain of SARS-CoV and SARS-CoV-2. Even though ACE2 is distributed in various organs, SARS-CoV-2 tends to infect a small number of organs compared to SARS-CoV [134]. Integrins are heterodimeric proteins (α and β subunits) on the cell membrane. Many integrins can recognize RGD and KGD motifs. RGD-recognizing integrins are αVβ1, αVβ3, αVβ5, αVβ6, αVβ8, αMβ2, αLβ2, and α3β1. KGD can be detected by αIIbβ3, αVβ5, αVβ6, and αVβ8 [134]. Integrins have been found to play a proviral function in SARS-CoV-2 entry. The RGD/KGD motif can be found in both the spike protein and its ACE2 receptor. Both SARS-CoV and SARS-CoV-2 entry are inhibited by integrins [134].

RGD/KGD integrin-binding motifs have been found in a variety of coronaviruses, including SARS-CoV and SARS-CoV-2 [135]. This KGD motif is in the exposed small loop in the open and closed ACE2 dimer structure, indicating that integrin can interact with ACE2 and S protein individually through this motif. Integrin interacts with ACE2 via its KGD motif, which contains K353, a key residue for spike recognition. As integrin interaction with the spike prevents the ACE2 RBM from detecting S RBD, viral entry is blocked [136].

**Figure 3:**
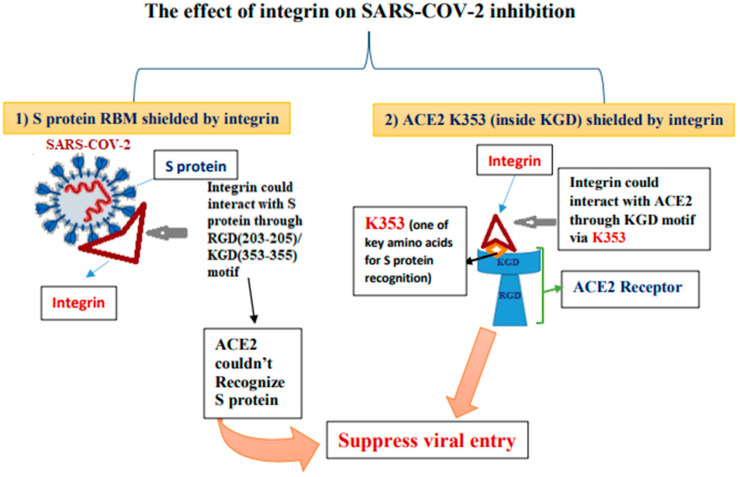
The critical inhibitory role of integrin in SARS-CoV-2.

The SARS-CoV-2 S protein requires an RGD integrin-binding motif, even though the RGD motif is not seen in other coronaviruses [135]. Additionally, by binding to integrins, the RGD motif acquired by SARS-CoV-2 promotes viral entry, thereby enhancing the transmission pathway. However, some studies found that integrins recognize both RGD and KGD motifs. The RGD motif is recognized by more types of integrins than the KGD motif [134,135,136]. When integrins attach to the S protein, the stereo-hindrance effect prevents S from reaching ACE2. The significant transmission efficiency might be associated with the key residues’ changes in the SARS-CoV-2 S protein and their equivalent receptors. The RGD motif is a cell attachment region that can detect distinct epithelial cell integrins to improve cell adherence and virus internalization through activation of transducing pathways involving phosphatidylinositol-3 kinase (PI-3K) or mitogen-activated protein kinase (MAPK) [136]. See Figure 3 for an illustrative description.

### 4.3. Glucose-Regulated Protein 78 (GRP78)

Glucose-regulated protein 78 (GRP78), known as heat shock protein A5 (HSPA5) and the binding immunoglobulin protein (BiP), is an integral endoplasmic reticulum (ER) chaperone protein engaged in the continuation and protein shadowing of unfolded protein response (UPR). The UPR is activated as a cell stress response initiated by the accumulation of unfolded or improperly folded proteins [100,137,138]. GRP78 is normally found in the ER lumen, where it attaches to and inactivates three enzymes involved in differentiation and cell death, including the protein kinase RNA-like endoplasmic reticulum kinase (PERK), activating transcription factor 6 (ATF6), and inositol-requiring enzyme 1 (IRE1) [139]. Accumulation of unfolded proteins results in GRP78 activation and the release of ATF6, PERK, and IRE1. The activation of enzymes results in protein synthesis and refolding enhancement. GRP78 overexpression is triggered by cell stress, which increases the likelihood of GRP78 escaping ER retention and moving to other cellular compartments [140]. Once GRP78 translocates to the cell membrane, it uses its substrate-binding domain (SBD) for some pathogen recognition and mediates its entry into the host cell [141,142,143,144]. This has been reported in many viruses, such as MERS-CoV, Ebola virus, Dengue virus, Japanese Encephalitis virus, Coxsackievirus A9, Zika virus, and Borna disease virus [92,140,145,146,147]. GRP78 has also been investigated as a potential viral entry point for SARS-CoV-2 by binding to motifs on the virus’s spike protein [100,142].

SARS-CoV-2 spike protein binds to GRP78 upon cell stress. The binding site was predicted in silico using the similarity between the cyclic peptide (Pep42), which has a specific binding affinity against GRP78 over cancerous cells [148]. This has declared the involvement of the SBDβ of GRP78 in the SARS-CoV-2 attachment and cell entry [100,142]. In addition, this has revealed that the fusion is favorable between the cyclic regions III (C391-C525) and IV (C480-C488) of GRP78 SBDβ and the spike [106,142]. As inhibiting the interface between the SARS-CoV-2 spike protein and the GRP78 host-cell receptor reduces the rate of viral infection, developing a SARS-CoV-2 spike protein vaccine would almost certainly prevent viral infection [100].

Gene expression of GRP78 and serum concentrations were enhanced in infections caused by the SARS-CoV-2 virus [142]. Damaged airway epithelial cells are thought to express GRP78 in response to severe pulmonary trauma and injury occurred during SARS-CoV-2 infection [142,149], which can cause significant inflammation in COVID-19 patients. GRP78 has been identified as a danger-associated molecular pattern (DAMP) for the toll-like receptors TLR2 [150] and TLR3 [151] and may cause increased inflammation in COVID-19 patients [142,149].

GRP78 mRNA levels were shown to be four times more likely in the blood of COVID-19 (+ve) pneumonia patients than COVID-19 (−ve) pneumonia patients, suggesting that GRP78 could be a potential molecular target for developing COVID-19 treatment. A total of 409 compounds with potential GRP78 inhibition properties were identified [129,142].

As GRP78 SBDβ has a strong affinity for hydrophobic regions, hydrophobic compounds would have a high affinity for GRP78 SBDβ, and may therefore hypothetically compete with virus spike recognition. In silico studies have suggested many compounds and peptides as potential inhibitors of GRP78, such as zilucoplan; obinepitide; corticorelin ovine triflutate; phytoestrogens; caffeic acid phenethyl ester; hydroxytyrosol; cinnamaldehyde; thymoquinone; and chlorogenic, linolenic, palmitic, cis-p-coumaric and caffeic acids [106,137].

Cross-vaccination is predicted to work using the SARS-CoV-2 spike regarding the previous human coronaviruses’ strains HKU1, 229E, NL63, and OC43. This is based on the SARS-CoV-2 spike’s GRP78 binding region (C480–C488) being conserved [152,153]. Furthermore, it has been reported that the cell-surface GRP78 contribution to SARS-CoV-2 spike recognition is enhanced in the new virus variants of the virus (VOC-202012/01, 501.V2, and B.1.1.248 lineage) with the mutations K417N, E484K, and N501Y [154,155]. This emphasizes the need to address GRP78, the viral entry mechanism, and the host-cell identification in future studies.

### 4.4. Toll-like Receptors 

The first-line protection against infectious pathogens is promoted by the innate immune system [149,156]. A superfamily of germline-coded proteins known as pattern recognition receptors (PRR) is crucial to innate immunity. Some of the PRRs, toll-like receptors (TLRs), are essential proteins that enable host regulation by recognizing external and self-molecular patterns [157,158]. TLRs are type I transmembrane glycoproteins with three structural components: an N-terminal intracellular toll-interleukin 1 receptor domain (necessary for signal transduction), a central transmembrane domain, and an extracellular C-terminus abundant with leucine repeats (which gives diversity to the individual TLRs) [159,160,161]. TLRs are each composed of an ectodomain with leucine-rich repeats (LRRs) and a cytoplasmic domain with a Toll/IL-1R homology (TIR) domain. The TLR family in human beings consists of ten members (TLR1–TLR10). TLRs can recognize a range of pathogen-associated molecular patterns (PAMPs), leading to the provoking of a robust inflammatory response to neutralize and remove invasive pathogens. Each TLR may recognize a specific set of ligands [157,158]. TLR1, TLR2, TLR4, TLR5, TLR6, and TLR10 are cell membrane exposed TLRs, whereas TLR3, TLR7, TLR8, TLR9, TLR11, TLR12, and TLR13 are endosomal TLRs [158,162].

Taking into account that the innate immune system relies on PRRs, which detect PAMPs [163], TLRs react to DAMP, independent of infection, and they are released by weakened, stressed, or necrotic cells [164,165]. The interaction of adaptor molecules containing the Toll/interleukin-1 receptor’s (TIR) structurally conserved domain is a crucial component of TLR signaling. The interaction of a TLR’s TIR domain with TRIF, myeloid differentiation factor 88 (MyD88), TIRAP, or TRAM contributes to its specificity. TLR signaling is classified into two types: MyD88-dependent and TRIF-dependent. MyD88 is used for signaling by TLR2, TLR5, TLR7, TLR78, and TLR9. TLR3 and TLR4 both recruit TRIF. The Myddosome is formed when MyD88 interacts with IRAK kinase family members. TRIF-dependent pathways involve Toll/IL-1 receptor domain-containing adaptors. Activated tyrosine kinase interacts with MyD88 and TRIF to stimulate the activation of MyD88-dependent and TRIF-dependent routes, increasing the generation of inflammatory cytokines and type I interferons (IFNs) [162,166,167]; the TRIF-dependent pathway is universal among the TLRs, regardless of the activating ligand [105,168]. To eliminate invading pathogens, signaling pathways produce antimicrobial peptides, proinflammatory cytokines, interferons, and chemokines [169,170].

## 5. SARS-CoV-2 Entry Receptors and Potential Therapeutic Targets

### 5.1. TLR1/2/6 in Proinflammatory Responses

The TLR2 receptor, which recognizes bacterial lipopeptides (LP), collaborates to form functional heterodimers with either TLR1 or TLR6 to mediate intracellular signaling [157,171,172]. TLR2 is regulated in chronic obstructive pulmonary disease (COPD) and predominantly detects invasive Gram-positive bacteria, mycobacteria, and fungi [173,174,175,176]. TLR2 heterodimers with either TLR1 or TLR6 enhanced proinflammatory responses during viral infection by identifying viral glycoproteins [177,178]. This implies a limited function for antiviral immunity [179]. The immunopathological functions played by TLR1 and TLR6 during SARS-CoV-2 infection remain to be clarified [178]. However, increased levels of TLR2 with either TLR1 or TLR6 DAMPs, including beta-defensin-3, named TLR1/2, and the high-mobility group box-1 (HMGB1), named TLR1/2/6, were recorded in peripheral blood mononuclear cells and serum obtained from COVID-19 patients [180,181,182]. The direct binding between DAMPs and the corresponding TLRs can trigger TLR-mediated inflammatory reactions, analogous to that induced by PAMP recognition [164]. Consequently, TLR1/2/6 activation and its consequent signal transduction may play a role in explaining the immunopathological symptoms observed by COVID-19 patients in clinical settings.

### 5.2. SARS-CoV-2 Infection and TLR3 Role in Antiviral Immunity

TLR3 is required for antiviral immunity because it recognizes and communicates with viral PAMPs, such as double-stranded ribonucleic acid (dsRNA) generated by positive sense-strand RNA and DNA viruses during viral replication [150,183], small interference RNA [151], and inadequate stem structures in single-stranded RNA [184]. Liberated cellular debris, besides the cytoplasmic nucleotides (messenger RNA and dsRNA) and GRP78, activates TLR3 DAMPs from host cells [151,185,186]. TLR3 is unique in that it is the only TLR that interacts solely with TRIF, activating both NF-κβ and interferon-regulatory factor-3 and 7 [150]. This interaction causes pro-inflammatory molecules to be released, such as IL-1β, IL-6, IL-8, and TNF-α, found in the immunopathological screening of COVID-19 patients [112,187]. Direct communication between TLR3 and the SARS-CoV-2 S protein has yet to be explained. TLR3 may recognize SARS-CoV-2 products released during viral replication, indicating that TLR3 may be a therapeutic target that, when activated, may increase antiviral immune responses, decrease viral loads, and promote SARS-CoV-2 blockage [188].

### 5.3. TLR4 Inhibition and SARS-CoV-2 Entry

TLR4 recognizes lipopolysaccharide (LPS) of bacteria and its activation generally results in the production of chemokines and pro-inflammatory cytokines [189]. Due to the physiological characterization of LPS, the TLR4 receptor is responsible for Gram-negative bacterial immunity [188,190]. TLR4 activation and interaction with viral fusion proteins and glycoproteins, such as those seen in respiratory viruses, have been described [191,192]. TLR4 can react to a variety of DAMPs originating from the host, which have been linked to increased and uncontrolled inflammation in autoimmune illnesses and chronic inflammatory disorders [193,194]. TLR4-mediated inflammation that is unregulated has been associated with immunopathological effects in COVID-19 patients [194]. In computational studies investigating the TLR-binding efficacy of S protein have demonstrated that TRL4 has the highest affinity for the S1 domain of the S protein [195]. As TLR4’s ability to suppress pathogens could constitute a novel viral entry route for SARS-CoV-2, TLR4 inhibition as a potential treatment in COVID-19 infection should be examined.

### 5.4. TLR5 as a Potential SARS-CoV-2 Vaccine Target

During vaccine development and to enhance the vaccine efficacy by tailoring the immune responses, a potent immunomodulatory agent called flagellin, which is a structural whip-like filament dependent on microtubules, has been used as an adjuvant component [196,197] due to its ability to influence pathogenic virulence to enable locomotion in motile Gram-negative and positive bacteria [165,198]. The interaction of flagellin with TLR5 leads to subsequent NF-kβ motivated inflammation through enrolment of MyD88 and has been shown to be an effective immunomodulatory agent [157,158,199,200,201]. The use of flagellin to target TLR5 in the creation of vaccines against viral infections has been studied. However, the interaction of TLR5 with SARS-CoV-2 needs to be investigated. In silico studies showed positive energy for TLR5 and S protein of SARS-CoV-2, indicating a possible association [195].

### 5.5. TLR7 and TLR8 Role in SARS-CoV-2 Infection

Toll-like receptors 7/8 (TLR7/8) are pattern recognition receptors (PRR) located on intracellular organelles that produce antiviral immunity by recognizing the viral single-stranded RNA (ssRNA) and releasing cytokines, chemokines, IFN-α, IFN-β, and IFN-λ as pro-inflammatory mechanisms [202,203]. Studies have demonstrated TLR7/8′s role in reducing viral replication in HIV-1 [204], influenza [195], and MERS-CoV [205]. When viral ssRNA binds to TLR7/8 upon viral entry, antiviral immunity is activated. The SARS-CoV-2 genome has shown more ssRNA segments that TLR7/8 can detect than the SARS-CoV genome, suggesting SARS-CoV-2 causes innate immune hyperactivation [206]. This observation suggested a strong pro-inflammatory response via TLR7/8 recognition. On the other hand, a larger number of SARS-CoV-2 fragments that TLR7/8 identified suggested that rapid release of type I IFNs by TLR7/8 influences the severity of SARS-CoV-2 by changing dendritic Cell (DC) growth, maturation, and apoptosis, and virus-specific cytotoxic responses produced by T lymphocytes and cytotoxicity of natural killer cells [206]. As DC function has been shown to be reduced, attempts to reverse this negative effect may be effective in Covid-19 treatment [207]. COVID-19 patients showed increased blood levels of pro-inflammatory cytokines and chemokines, which are produced by the TLR7/8 pathways [202]. This could be attributed to an increase in TLR7/8 recognizing antiphospholipid antibodies (aPL) (a TLR7/8 activating DAMP) in COVID-19 patients [187,208,209]. TLR7 and TLR8 activation could be employed to improve viral immunity as a potential therapeutic therapy. Based on data analysis collected from mice models treated with imiquimod following influenza A infection, imiquimod, a dual TLR7/8 agonist, has been proposed as a viable treatment for COVID-19 patients [210]. Direct infusion of imiquimod into the lungs lowers viral multiplication, avoids pulmonary inflammation and leukocyte infiltration; protects against pulmonary dysfunction worsening; and elevates pulmonary immunoglobulins and bronchiole fluid antibodies (such as IgG1, IgG2a, IgE, and IgM) [211]. Due to its role in increasing antigen-specific antibody production and enhancing the immune response for viral clearance, imiquimod could be used both for COVID-19 therapeutic treatment and as an adjuvant in the SARS-CoV-2 vaccine [212,213].

### 5.6. C-Lectin Type Receptors Involved with SARS-CoV-2

C-type lectin receptors (CLRs) are a large family of transmembrane-soluble pattern recognition receptors that contain one or more conserved carbohydrate-recognition domains [214,215]. Such receptors can help in the calcium-dependent recognition of glycosylation marks present on pathogens’ proteins [216]. CLRs interact with mannose, fucose, and glucan mono- and polysaccharide structures to identify infections [217]. PAMP recognition by CLRs results in pathogen uptake, breakdown, and antigen presentation [218]. CLRs can as well connect with other PRRs, such as TLRs, allowing for the strengthening or weakening of innate immunity inflammatory responses by increasing or decreasing receptor activation and signal transduction [219,220]. In vitro study models have demonstrated a direct relationship between selective CLRs and SARS-CoV-2 spike protein mannosylated and N- and O-glycans [221].

## 6. Conclusions

The coronavirus disease COVID-19, caused by the SARS-CoV-2 virus, spreads mainly through person-to-person contact. SARS-CoV-2 is one of seven identified human coronaviruses that can cause serious illnesses. SARS-CoV-2 can trigger a respiratory tract infection, ranging from mild to deadly, and can cause respiratory failure, septic shock, pneumonia, heart, and liver complications, and may lead to death.

We covered the history and progression of human coronaviruses in this paper and the various host-cell receptors that may be engaged in the viral entry mechanism, showing that the SARS-CoV-2 virus can use multiple receptors to enter the host-cells. Understanding the mechanism of SARS-CoV-2 infection requires determining the pathway through which the virus components bind to host-cell receptors. The information gathered in this study can be used as a guided tool to investigate how different cell types interact with the SARS-CoV-2 virus, while supported experimental investigations are required to explain the susceptibility differences to the viral infection. Afterward, we could ultimately be able to explain why some people are more susceptible to SARS-CoV-2 infection than others. In addition, it could help researchers understand how to specifically target the SARS-CoV-2 virus with drugs and immunotherapies to treat COVID-19 symptoms and improve the vaccine development research pipeline to prevent the disease.

## Figures and Tables

**Table 1:** Mortality rate of pre-existing cardiovascular disease (CVD) as a risk factor in COVID-19 patients.

Publication	Publication Date	Total N of Cases	N of Deceased Cases	N of CVD Cases	N of CVD Deceased	CVD Fatality (N of CVD Deceased/N of CVD) %	Mortality Rate (N of CVD Deceased/N Deceased) %
Q. Ruan et al. [118]	March 2020	150	68	25	20	80	29.41
F. Zhou et al. [119]	March 2020	191	54	15	13	87	24.07
RM. Inciardi et al. [120]	May 2020	99	26	53	19	36	73.08
ES. Rosenberg et al. [121]	May 2020	1438	292	438	136	31	46.58
F. San Roman et al. [122]	June 2020	522	130	68	43	63	33.08
G. Grasselli et al. [123]	July 2020	3988	1926	533	342	64	17.76
S. Gupta et al. [124]	July 2020	2215	784	288	130	45	16.58
B. Thakur et al. [125]	April 2021	364	299	58	51	87.93	17.06
K. Nakamichi et al. [126]	February 2021	190	14	34	9	26.47	64.29
K. O’Gallagher et al. [127]	July 2021	1721	438	349	130	37.25	29.68

## Data Availability

Not applicable.
